# Association of Non-traditional Indicators of Readers’ Engagement With Traditional Dissemination Metrics of COVID-19-Related Research

**DOI:** 10.7759/cureus.34238

**Published:** 2023-01-26

**Authors:** Faran Ahmad, Matthew Merwin, Abbis H Jaffri, Bryan Krajicek

**Affiliations:** 1 Infectious Diseases-Critical Care Medicine, Creighton University Medical Center, Omaha, USA; 2 Critical Care Medicine, Veterans Affairs Medical Center, Omaha, USA; 3 Department of Medicine, Creighton University School of Medicine, Omaha, USA; 4 Research and Development, Creighton University School of Medicine, St. Joseph's Hospital and Medical Center, Omaha, USA; 5 Pulmonary and Critical Care Medicine, Creighton University School of Medicine, Omaha, USA

**Keywords:** sars-cov-2, journal impact factor, citation metrics, altmetric analysis, altmetric attention score

## Abstract

Introduction: Researchers are increasingly interested in appraising the impact of their research work, which eventually drives public perception. The overall impact of a study can only be gauged if we consider both traditional and non-traditional dissemination patterns. Hence, we preferred to study the association between the non-traditional reader engagement metrics and traditional dissemination metrics in relation to coronavirus disease 2019 (COVID-19)-related research published in five high-impact peer-reviewed medical journals.

Method: This observational study was conducted using data sourced from Altmetric, including the Altmetric attention score (AAS), an aggregate score of an article’s dissemination. New England Journal of Medicine (NEJM), Lancet Infectious Diseases, Clinical Infectious Diseases (CID), Chest Journal (CHEST), and Journal of the American Medical Association (JAMA) were included in the study based on the prevalence of COVID-19-related original research published in each of them. The number of citations was framed as the reference for traditional metrics. To avoid artificial variance, data were collected on the same day, November 13, 2022. Correlational analyses were performed using the Pearson correlation coefficient using Minitab 17 (Minitab Inc., State College, PA). The relationship between the variables was considered very weak if r<0.3, weak if r: 0.3 to 0.5, moderate if r: 0.5 to 0.7, and strong for r>0.7.

Results: We found a very weak correlation between citations and AAS for Clinical Infectious Diseases, Lancet Infectious Diseases, and CHEST, whereas the correlation was moderate for NEJM and JAMA. The correlation between citations and Twitter mentions was very weak for Clinical Infectious Disease, Lancet Infectious Disease, and CHEST, but it improved for NEJM and JAMA. There was a very weak correlation between citations and news mentions for Clinical Infectious Diseases, Lancet Infectious Diseases, and CHEST.

Conclusion: Our study highlights that the traditional indicator, i.e., citation has a very weak to moderate correlation with the AAS and it doesn’t capture the entire influence of a research publication. Also, the current method of determining a journal's impact factor doesn’t take this disparity into consideration. Hence, there needs to have a more inclusive strategy to define the impact of scientific research on the general population in real-time.

## Introduction

As of 2021, nearly half of the American social media users reported that they often get their news from social media [1]. Dissemination of original research should follow similar emerging trends and hence allow greater public access to the research. Researchers and institutions are increasingly interested in appraising the impact of their research work. As the coronavirus disease 2019 (COVID-19) pandemic progressed, we understood that public access and interaction with the original research was necessary to comprehend the best prevention practices. Interestingly, in 2020, the top five most discussed research articles across 20 different educational disciplines were all related to COVID-19 [2]. Our study focused on examining the association between various alternative indicators of readers’ engagement with COVID-19 original research and traditional dissemination metrics of research published in the five high-impact peer-reviewed medical journals.

## Materials and methods

This cross-sectional study was conducted using data sourced from Altmetric, including the Altmetric attention score (AAS), an aggregate score of an article’s dissemination on the internet that takes into consideration mentions from news sources, blogs, social media, and citations within the scientific community [3]. For the purpose of this study, AAS and its sub-components were used to gauge metrics of non-traditional research dissemination. The access was granted to the Altmetric Explorer by Altmetric and received institutional review board (IRB) approval from Creighton University, Omaha, Nebraska, Approval number: 2003168-01. A PubMed query was conducted within the Altmetric Explorer to identify the COVID-19-related research articles. New England Journal of Medicine (NEJM), Lancet Infectious Diseases, Clinical Infectious Diseases (CID), Chest Journal (CHEST), and Journal of the American Medical Association (JAMA) were included in the study based on their impact factors and the prevalence of COVID-19-related original research published in each of them. Key words included COVID-19, COVID19, COVID 19, SARS-CoV2, SARS-CoV-2, coronavirus-19, coronavirus19, and coronavirus 19. Articles not tracked by Altmetric were excluded. A correlation between traditional and non-traditional metrics was established for each journal to elaborate on their dissemination pattern. The number of citations was framed as the reference for traditional metrics. To avoid artificial variance, data were collected on the same day, November 13, 2022. All the COVID-19-related studies from January 1, 2020, till the date of data collection, were included. Correlational analyses were performed using the Pearson correlation coefficient using Minitab 17 (Minitab Inc., State College, PA). The relationship between the two variables was considered very weak if r<0.3, weak if r: 0.3 to 0.5, moderate if r: 0.5 to 0.7, and strong if r>0.7.

## Results

We found a very weak correlation between citations and AAS for Clinical Infectious Diseases (r=0.21), Lancet Infectious Diseases (r=0.22), and CHEST (r=0.24), whereas the correlation was moderate for NEJM (r=0.52) and JAMA (r=0.5). The correlation between citations and Mendeley (Elsevier, London, UK), a reference manager, was strong for all the journals, with r>0.7 (Table 1). Moreover, the correlation between citations and Twitter mentions was very weak for Clinical Infectious Diseases (r=0.12), Lancet Infectious Diseases (r=0.14), and CHEST (r=0.22) but it was higher for NEJM (r=0.47) and JAMA (r=0.4) (Table 2). There was a very weak correlation between citations and news mentions for Clinical Infectious Diseases (r=0.19), Lancet Infectious Diseases (r=0.25), and CHEST (r=0.15), whereas this correlation was moderate for NEJM and JAMA (r=0.58 for both) (Table 2). The COVID-19-related article with the highest AAS was published on 12/13/2020 in NEJM with a score of 30136, and the COVID-19-article with the highest number of citations (20808) was also published in NEJM on 4/30/2020 (Appendix 1, Table 4). Among these five peer-reviewed journals, Clinical Infectious Diseases had the most number of COVID-19-related publications from January 1, 2020, to the date of data extraction (November 13, 2022). 

**Table 1:** Total COVID-19-related articles in the selected journals from January 1, 2020, till the day of data collection (November 13, 2022), and the highest Altmetric attention scores (AAS) on an article. NEJM: New England Journal of Medicine, CHEST: Chest Journal, JAMA: Journal of the American Medical Association.

Journals	Total COVID-19-related articles till the date of data collection (n)	Highest Altmetric attention scores on an article	Impact factor (based on 2021 Journal Citation Reports®, Clarivate 2022)
NEJM	618	30136	176.079
Clinical Infectious Diseases	1085	9971	9.079
Lancet Infectious Diseases	491	18921	71.421
CHEST	217	3278	11.393
JAMA	814	12973	157.335

**Table 2:** Correlation between traditional (citation) and non-traditional dissemination metrics (very weak if r<0.3, weak if r: 0.3 to 0.5, moderate if r: 0.5 to 0.7, and strong if r>0.7). NEJM: New England Journal of Medicine, JAMA: Journal of the American Medical Association.

Journals	Citations vs AAS (r)	Citations vs Mendeley (r)	Citations vs Twitter mentions (r)	Citations vs news mentions (r)
NEJM	0.52	0.97	0.47	0.58
Clinical Infectious Diseases	0.21	0.94	0.12	0.19
Lancet Infectious Diseases	0.22	0.92	0.14	0.25
Chest	0.24	0.86	0.22	0.15
JAMA	0.50	0.95	0.40	0.58

## Discussion

The AAS indicates the amount of attention a research work has received. It is based on an automated algorithm that takes into account the weighted count of the amount of attention a media source gets. This weighted count is based on the relative reach of the type of each source. Each media source has default weightings, e.g., a news portal gets a default weighting of eight as compared to a tweet having a default weighting of 0.25, just as a news mention will bring more attention to a topic as compared to a simple tweet [4]. Further details of these default weightings are reflected in Table 3. In addition, AAS further takes into account other factors, including duplicate tweets or tiered calculations for different types of news sources depending upon their dissemination and reliability. By blending all that useful information, AAS helps a researcher to gauge the dissemination of their research, identify new potential collaborators, and measure the impact of their work.

**Table 3:** The default weightings of each type of source.

News	8
Blogs	5
Policy documents (per source)	3
Patent citations	3
Wikipedia	3
Peer review (Publons, Pubpeer)	1
Google+ (not trackable since 2019, but historical data kept)	1
F1000	1
Syllabi (open syllabus)	1
Twitter (tweets and retweets)	0.25
Facebook (only a curated list of public pages)	0.25
Reddit	0.25
YouTube	0.25

Multiple prior studies have reported a positive correlation (weak to moderate) between citations and an Altmetric score, as reflected by our study [5-14]. However, our study results also highlight that although traditional indicators, i.e., citations do correlate with the AAS, they do not capture the entire impact of a research study or a researcher. There needs to have a more inclusive strategy to define the impact of landmark scientific research studies on the general population in real-time [15]. Also, the AAS provides immediate information regarding the dissemination statistics of an article as compared to the citations, which may take years to grow [16]. AAS provides real-time data on readership trends. This also highlights the area of improvement across the board to amplify the access of people outside of the scientific community to ever-evolving research and clinical trials. Both NEJM and JAMA have relatively more dissemination, as reflected by their higher AAS compared to Lancet Infectious Disease, CID, and CHEST. This signals a more significant momentum within NEJM and JAMA to improve the outreach of their respective journal articles.

We believe that by utilizing these alternative metrics of knowledge dissemination, journals can increase the acceptability of emerging research in the public and hence improve public behavioral practices as well as debunk the myths. However, all five journals have a close relationship between Mendeley's readership and citations. This relationship between Mendeley's readership and citations is consistent with previous studies as well [17]. Hence, Mendeley's readership numbers may serve as a proxy for citations, as these readers are largely members of the scientific community and are therefore more likely to cite a study in future work [18].

There are some limitations of this study. Altmetric Explorer itself is not that comprehensive, and the AAS score serves only as a proxy for reader engagement but does not tell us if that changed the public's perspective. Also, there is no “good” AAS, as it simply reflects the extent of dissemination. It cannot differentiate if a research article got a high attention score because of negative coverage in the news or mentions on social media, resulting in a negative impact on public perception. Future directions may include more comprehensive studies to elaborate on the role of alternative metrics of research dissemination and hence highlight the probability of utilizing these matrices to evaluate researchers’ impact.

## Conclusions

While current methods of gauging the impact of scientific research largely rely on citations, which will take years to grow, ongoing scoring systems like AAS may provide real-time data on readership trends. Logically, non-traditional dissemination metrics, including AAS, may be combined with the current calculation methodology to define the real-time impact of a researcher and their scientific work.

## Appendices

Appendix 1 

**Table 4:** Altmetric attention scores, number of citations, and the details of the COVID-19-related articles published in NEJM from January 1, 2022, till the data extraction date (November 13, 2022).

Altmetric attention score	Title	Publication date	DOI	Number of citations
30136	Safety and Efficacy of the BNT162b2 mRNA COVID-19 Vaccine	12/31/2020	10.1056/nejmoa2034577	8447
24770	Aerosol and Surface Stability of SARS-CoV-2 as Compared with SARS-CoV-1	4/16/2020	10.1056/nejmc2004973	6495
19816	Preliminary Findings of mRNA COVID-19 Vaccine Safety in Pregnant Persons	6/17/2021	10.1056/nejmoa2104983	478
16954	Effect of Early Treatment with Ivermectin among Patients with COVID-19	5/5/2022	10.1056/nejmoa2115869	48
16763	Effectiveness of COVID-19 Vaccines against the B.1.617.2 (Delta) Variant	8/12/2021	10.1056/nejmoa2108891	1836
14846	BNT162b2 mRNA COVID-19 Vaccine in a Nationwide Mass Vaccination Setting	4/15/2021	10.1056/nejmoa2101765	1720
14235	An mRNA Vaccine against SARS-CoV-2—Preliminary Report	11/12/2020	10.1056/nejmoa2022483	2266
13086	Remdesivir for the Treatment of COVID-19—Final Report	11/5/2020	10.1056/nejmoa2007764	4832
12996	COVID-19—Navigating the Uncharted	3/26/2020	10.1056/nejme2002387	1218
12989	Safety of the BNT162b2 mRNA COVID-19 Vaccine in a Nationwide Setting	9/16/2021	10.1056/nejmoa2110475	453
10843	A Randomized Trial of Hydroxychloroquine as Postexposure Prophylaxis for COVID-19	8/6/2020	10.1056/nejmoa2016638	996
10705	Facial Masking for COVID-19—Potential for "Variolation" as We Await a Vaccine	10/29/2020	10.1056/nejmp2026913	171
10627	Efficacy and Safety of the mRNA-1273 SARS-CoV-2 Vaccine	2/4/2021	10.1056/nejmoa2035389	5663
10298	Universal Masking in Hospitals in the COVID-19 Era	5/21/2020	10.1056/nejmp2006372	208
10037	Clinical Characteristics of Coronavirus Disease 2019 in China	4/30/2020	10.1056/nejmoa2002032	20808
9411	Dexamethasone in Hospitalized Patients with COVID-19	2/25/2021	10.1056/nejmoa2021436	7104
9110	Universal Screening for SARS-CoV-2 in Women Admitted for Delivery	5/28/2020	10.1056/nejmc2009316	856
8637	Open Schools, COVID-19, and Child and Teacher Morbidity in Sweden	2/18/2021	10.1056/nejmc2026670	58
8445	A Trial of Lopinavir–Ritonavir in Adults Hospitalized with Severe COVID-19	5/7/2020	10.1056/nejmoa2001282	3977
8416	Evaluation of the BNT162b2 COVID-19 Vaccine in Children 5 to 11 Years of Age	1/6/2022	10.1056/nejmoa2116298	266
8352	Effectiveness of an Inactivated SARS-CoV-2 Vaccine in Chile	9/2/2021	10.1056/nejmoa2107715	469
8332	Protection of BNT162b2 Vaccine Booster against COVID-19 in Israel	10/7/2021	10.1056/nejmoa2114255	725
8260	Compassionate Use of Remdesivir for Patients with Severe COVID-19	6/11/2020	10.1056/nejmoa2007016	2099
8248	COVID-19 Breakthrough Infections in Vaccinated Health Care Workers	10/14/2021	10.1056/nejmoa2109072	839
8035	Observational Study of Hydroxychloroquine in Hospitalized Patients with COVID-19	6/18/2020	10.1056/nejmoa2012410	1268
7989	Efficacy of the ChAdOx1 nCoV-19 COVID-19 Vaccine against the B.1.351 Variant	5/20/2021	10.1056/nejmoa2102214	957
7865	Duration of Shedding of Culturable Virus in SARS-CoV-2 Omicron (BA.1) Infection	7/21/2022	10.1056/nejmc2202092	30
7540	Spread of SARS-CoV-2 in the Icelandic Population	6/11/2020	10.1056/nejmoa2006100	1104
7531	Lifting Universal Masking in Schools—COVID-19 Incidence among Students and Staff	11/9/2022	10.1056/nejmoa2211029	1
7463	Safety, Immunogenicity, and Efficacy of the BNT162b2 COVID-19 Vaccine in Adolescents	7/15/2021	10.1056/nejmoa2107456	479
7452	Effectiveness of the BNT162b2 COVID-19 Vaccine against the B.1.1.7 and B.1.351 Variants	7/8/2021	10.1056/nejmc2104974	765
7028	Effects of Previous Infection and Vaccination on Symptomatic Omicron Infections	7/7/2022	10.1056/nejmoa2203965	91
6973	Severity of SARS-CoV-2 Reinfections as Compared with Primary Infections	12/23/2021	10.1056/nejmc2108120	58
6696	Genomewide Association Study of Severe COVID-19 with Respiratory Failure	10/15/2020	10.1056/nejmoa2020283	1327
6570	BNT162b2 Vaccine Booster and Mortality Due to COVID-19	12/23/2021	10.1056/nejmoa2115624	186
6552	Protection by a Fourth Dose of BNT162b2 against Omicron in Israel	5/5/2022	10.1056/nejmoa2201570	132
6534	Myocarditis after BNT162b2 mRNA Vaccine against COVID-19 in Israel	12/2/2021	10.1056/nejmoa2109730	292
6311	Thrombotic Thrombocytopenia after ChAdOx1 nCov-19 Vaccination	6/3/2021	10.1056/nejmoa2104840	1417
6282	Effectiveness of BNT162b2 Vaccine against Omicron in Children 5 to 11 Years of Age	8/11/2022	10.1056/nejmoa2203209	12
6179	Hydroxychloroquine with or without Azithromycin in Mild-to-Moderate COVID-19	11/19/2020	10.1056/nejmoa2019014	801
6174	Asymptomatic Transmission, the Achilles' Heel of Current Strategies to Control COVID-19	5/28/2020	10.1056/nejme2009758	934
6057	Efficacy of a Fourth Dose of COVID-19 mRNA Vaccine against Omicron	4/7/2022	10.1056/nejmc2202542	129
5884	Effect of Vaccination on Household Transmission of SARS-CoV-2 in England	8/19/2021	10.1056/nejmc2107717	231
5675	Responding to COVID-19—A Once-in-a-Century Pandemic?	4/30/2020	10.1056/nejmp2003762	719
5642	Protecting Olympic Participants from COVID-19—The Urgent Need for a Risk-Management Approach	7/1/2021	10.1056/nejmp2108567	22
5560	Prevention and Attenuation of COVID-19 with the BNT162b2 and mRNA-1273 Vaccines	7/22/2021	10.1056/nejmoa2107058	284
5493	COVID-19 Vaccine Effectiveness against the Omicron (B.1.1.529) Variant	4/21/2022	10.1056/nejmoa2119451	713
5467	Antibody Persistence through 6 Months after the Second Dose of mRNA-1273 Vaccine for COVID-19	6/10/2021	10.1056/nejmc2103916	480
5422	SARS-CoV-2 Infection in Children	4/23/2020	10.1056/nejmc2005073	1810
5332	Rethinking COVID-19 Test Sensitivity—A Strategy for Containment	11/26/2020	10.1056/nejmp2025631	572
5325	SARS-CoV-2 Viral Load in Upper Respiratory Specimens of Infected Patients	2/19/2020	10.1056/nejmc2001737	3843
5258	Successful Elimination of COVID-19 Transmission in New Zealand	8/20/2020	10.1056/nejmc2025203	231
5169	Myocarditis after COVID-19 Vaccination in a Large Health Care Organization	12/2/2021	10.1056/nejmoa2110737	298
5068	Neutralization Escape by SARS-CoV-2 Omicron Subvariants BA.2.12.1, BA.4, and BA.5	7/7/2022	10.1056/nejmc2206576	132
4953	Protection and Waning of Natural and Hybrid Immunity to SARS-CoV-2	6/9/2022	10.1056/nejmoa2118946	74
4904	Antibody Status and Incidence of SARS-CoV-2 Infection in Health Care Workers	2/11/2021	10.1056/nejmoa2034545	701
4885	Resurgence of SARS-CoV-2 Infection in a Highly Vaccinated Health System Workforce	9/30/2021	10.1056/nejmc2112981	111
4668	Fair Allocation of Scarce Medical Resources in the Time of COVID-19	5/21/2020	10.1056/nejmsb2005114	1959
4625	Safety and Efficacy of the BNT162b2 mRNA COVID-19 Vaccine through 6 Months	11/4/2021	10.1056/nejmoa2110345	662
4478	Safety and Efficacy of the BNT162b2 mRNA COVID-19 Vaccine	4/22/2021	10.1056/nejmc2036242	212
4417	Durability of Responses after SARS-CoV-2 mRNA-1273 Vaccination	1/7/2021	10.1056/nejmc2032195	595
4343	Antibody Responses in Seropositive Persons after a Single Dose of SARS-CoV-2 mRNA Vaccine	4/8/2021	10.1056/nejmc2101667	559
4277	Waning of BNT162b2 Vaccine Protection against SARS-CoV-2 Infection in Qatar	12/9/2021	10.1056/nejmoa2114114	480
4266	Waning Immunity after the BNT162b2 Vaccine in Israel	12/9/2021	10.1056/nejmoa2114228	584
4220	Persistence and Evolution of SARS-CoV-2 in an Immunocompromised Host	12/3/2020	10.1056/nejmc2031364	808
4214	Protection against the Omicron Variant from Previous SARS-CoV-2 Infection	3/31/2022	10.1056/nejmc2200133	172
4143	Large-Vessel Stroke as a Presenting Feature of COVID-19 in the Young	5/14/2020	10.1056/nejmc2009787	1625
4122	Transmission of SARS-CoV-2 in Domestic Cats	8/6/2020	10.1056/nejmc2013400	373
4054	Early Evidence of the Effect of SARS-CoV-2 Vaccine at One Medical Center	5/20/2021	10.1056/nejmc2102153	79
4045	Presymptomatic SARS-CoV-2 Infections and Transmission in a Skilled Nursing Facility	5/28/2020	10.1056/nejmoa2008457	1726
3997	Humoral Immune Response to SARS-CoV-2 in Iceland	10/29/2020	10.1056/nejmoa2026116	802
3967	Repurposed Antiviral Drugs for COVID-19—Interim WHO Solidarity Trial Results	2/11/2021	10.1056/nejmoa2023184	1696
3961	False Negative Tests for SARS-CoV-2 Infection—Challenges and Implications	8/6/2020	10.1056/nejmp2015897	681
3929	Safety and Efficacy of NVX-CoV2373 COVID-19 Vaccine	9/23/2021	10.1056/nejmoa2107659	444
3907	Oral Nirmatrelvir for High-Risk, Nonhospitalized Adults with COVID-19	4/14/2022	10.1056/nejmoa2118542	383
3888	Interim Results of a Phase 1–2a Trial of Ad26.COV2.S COVID-19 Vaccine	5/13/2021	10.1056/nejmoa2034201	802
3839	Efficacy and Safety of NVX-CoV2373 in Adults in the United States and Mexico	2/10/2022	10.1056/nejmoa2116185	106
3821	mRNA COVID-19 Vaccines in Pregnant Women	6/17/2021	10.1056/nejme2107070	18
3807	Thrombosis and Thrombocytopenia after ChAdOx1 nCoV-19 Vaccination	6/3/2021	10.1056/nejmoa2104882	931
3685	Cardiovascular Disease, Drug Therapy, and Mortality in COVID-19	6/18/2020	10.1056/nejmoa2007621	974
3678	Safety and Immunogenicity of Two RNA-Based COVID-19 Vaccine Candidates	12/17/2020	10.1056/nejmoa2027906	1682
3640	Retraction: Cardiovascular Disease, Drug Therapy, and Mortality in COVID-19. N Engl J Med. DOI: 10.1056/NEJMoa2007621.	6/25/2020	10.1056/nejmc2021225	408
3612	Efficacy of the mRNA-1273 SARS-CoV-2 Vaccine at Completion of Blinded Phase	11/4/2021	10.1056/nejmoa2113017	232
3583	Evaluation of the mRNA-1273 Vaccine against SARS-CoV-2 in Nonhuman Primates	10/15/2020	10.1056/nejmoa2024671	794
3537	Vaccine Breakthrough Infections with SARS-CoV-2 Variants	4/21/2021	10.1056/nejmoa2105000	521
3519	Delayed Large Local Reactions to mRNA-1273 Vaccine against SARS-CoV-2	4/1/2021	10.1056/nejmc2102131	194
3510	Toxic Effects from Ivermectin Use Associated with Prevention and Treatment of COVID-19	12/2/2021	10.1056/nejmc2114907	33
3440	Pulmonary Vascular Endothelialitis, Thrombosis, and Angiogenesis in COVID-19	7/9/2020	10.1056/nejmoa2015432	3662
3361	SARS-CoV-2 Transmission among Marine Recruits during Quarantine	12/17/2020	10.1056/nejmoa2029717	75
3347	BNT162b2 Protection against the Omicron Variant in Children and Adolescents	5/19/2022	10.1056/nejmoa2202826	62
3259	Nirmatrelvir Use and Severe COVID-19 Outcomes during the Omicron Surge	9/1/2022	10.1056/nejmoa2204919	19
3249	Rapid Decay of Anti-SARS-CoV-2 Antibodies in Persons with Mild COVID-19	9/10/2020	10.1056/nejmc2025179	915
3240	Evaluation of mRNA-1273 SARS-CoV-2 Vaccine in Adolescents	12/9/2021	10.1056/nejmoa2109522	165
3188	Early Remdesivir to Prevent Progression to Severe COVID-19 in Outpatients	1/27/2022	10.1056/nejmoa2116846	323
3174	Viral Dynamics of SARS-CoV-2 Variants in Vaccinated and Unvaccinated Persons	12/23/2021	10.1056/nejmc2102507	129
3147	Evaluation of mRNA-1273 Vaccine in Children 6 Months to 5 Years of Age	11/3/2022	10.1056/nejmoa2209367	0
3143	Effectiveness of BNT162b2 Vaccine against Critical COVID-19 in Adolescents	2/24/2022	10.1056/nejmoa2117995	76
3133	A Randomized Trial of Convalescent Plasma in COVID-19 Severe Pneumonia	2/18/2021	10.1056/nejmoa2031304	649
3075	Effect of Hydroxychloroquine in Hospitalized Patients with COVID-19	11/19/2020	10.1056/nejmoa2022926	846
3058	Safety and Efficacy of Single-Dose Ad26.COV2.S Vaccine against COVID-19	6/10/2021	10.1056/nejmoa2101544	1430
3003	SARS-CoV-2 Infection of Airway Cells	9/3/2020	10.1056/nejmicm2023328	15
2974	Multisystem Inflammatory Syndrome in U.S. Children and Adolescents	7/23/2020	10.1056/nejmoa2021680	1591
2951	Fourth Dose of BNT162b2 mRNA COVID-19 Vaccine in a Nationwide Setting	4/28/2022	10.1056/nejmoa2201688	72
2947	New-Onset Diabetes in COVID-19	8/20/2020	10.1056/nejmc2018688	504
2931	Saliva or Nasopharyngeal Swab Specimens for Detection of SARS-CoV-2	9/24/2020	10.1056/nejmc2016359	693
2880	Randomized Trial of Metformin, Ivermectin, and Fluvoxamine for COVID-19	8/18/2022	10.1056/nejmoa2201662	15
2857	Early High-Titer Plasma Therapy to Prevent Severe COVID-19 in Older Adults	2/18/2021	10.1056/nejmoa2033700	614
2840	SARS-CoV-2 Infection after Vaccination in Health Care Workers in California	5/6/2021	10.1056/nejmc2101927	199
2602	Protection against SARS-CoV-2 after COVID-19 Vaccination and Previous Infection	3/31/2022	10.1056/nejmoa2118691	155
2566	Effect of Vaccination on Transmission of SARS-CoV-2	10/28/2021	10.1056/nejmc2106757	110
2560	Effect of COVID-19 Vaccination on Transmission of Alpha and Delta Variants	2/24/2022	10.1056/nejmoa2116597	149
2538	Waning Immune Humoral Response to BNT162b2 COVID-19 Vaccine over 6 Months	12/9/2021	10.1056/nejmoa2114583	827
2537	Pan-Sarbecovirus Neutralizing Antibodies in BNT162b2-Immunized SARS-CoV-1 Survivors	10/7/2021	10.1056/nejmoa2108453	109
2537	COVID-19 Boosters—Where from Here?	4/28/2022	10.1056/nejme2203329	13
2534	COVID-19 Vaccination during Pregnancy and First-Trimester Miscarriage	11/18/2021	10.1056/nejmc2114466	55
2475	Baricitinib plus Remdesivir for Hospitalized Adults with COVID-19	3/4/2021	10.1056/nejmoa2031994	1009
2469	A Possible Role for Anti-idiotype Antibodies in SARS-CoV-2 Infection and Vaccination	1/27/2022	10.1056/nejmcibr2113694	32
2462	SARS-CoV-2 Neutralization with BNT162b2 Vaccine Dose 3	10/21/2021	10.1056/nejmc2113468	259
2457	Three Doses of an mRNA COVID-19 Vaccine in Solid-Organ Transplant Recipients	8/12/2021	10.1056/nejmc2108861	567
2444	Receipt of mRNA COVID-19 Vaccines and Risk of Spontaneous Abortion	10/14/2021	10.1056/nejmc2113891	87
2401	Subcutaneous REGEN-COV Antibody Combination to Prevent COVID-19	9/23/2021	10.1056/nejmoa2109682	268
2352	SARS-CoV-2 Omicron Variant Neutralization after mRNA-1273 Booster Vaccination	3/17/2022	10.1056/nejmc2119912	210
2336	A Cluster-Randomized Trial of Hydroxychloroquine for Prevention of COVID-19	2/4/2021	10.1056/nejmoa2021801	155
2330	Ensuring Uptake of Vaccines against SARS-CoV-2	6/26/2020	10.1056/nejmp2020926	67
2329	Safety and Immunogenicity of SARS-CoV-2 mRNA-1273 Vaccine in Older Adults	12/17/2020	10.1056/nejmoa2028436	987
2325	SARS-CoV-2 Neutralizing Antibody LY-CoV555 in Outpatients with COVID-19	1/21/2021	10.1056/nejmoa2029849	973
2321	Microvascular Injury in the Brains of Patients with COVID-19	2/4/2021	10.1056/nejmc2033369	277
2317	Protection against COVID-19 by BNT162b2 Booster across Age Groups	12/23/2021	10.1056/nejmoa2115926	133
2249	Neutralization Profile after Recovery from SARS-CoV-2 Omicron Infection	5/5/2022	10.1056/nejmc2201607	43
2187	Neurologic Features in Severe SARS-CoV-2 Infection	6/4/2020	10.1056/nejmc2008597	1832
2180	Molnupiravir for Oral Treatment of COVID-19 in Nonhospitalized Patients	2/10/2022	10.1056/nejmoa2116044	546
2079	Developing COVID-19 Vaccines at Pandemic Speed	5/21/2020	10.1056/nejmp2005630	1068
2027	Household Transmission of SARS-CoV-2 from Children and Adolescents	9/2/2021	10.1056/nejmc2031915	54
2026	COVID-19 in Critically Ill Patients in the Seattle Region—Case Series	5/21/2020	10.1056/nejmoa2004500	2007
2008	Guillain–Barré Syndrome Associated with SARS-CoV-2	6/25/2020	10.1056/nejmc2009191	968
2003	Risk of BA.5 Infection among Persons Exposed to Previous SARS-CoV-2 Variants	9/8/2022	10.1056/nejmc2209479	14
1975	Effectiveness of COVID-19 Vaccines in Ambulatory and Inpatient Care Settings	10/7/2021	10.1056/nejmoa2110362	224
1973	Pathologic Antibodies to Platelet Factor 4 after ChAdOx1 nCoV-19 Vaccination	4/16/2021	10.1056/nejmoa2105385	641
1942	A Bivalent Omicron-Containing Booster Vaccine against COVID-19	10/6/2022	10.1056/nejmoa2208343	31
1883	SARS-CoV-2 Vaccine–Induced Immune Thrombotic Thrombocytopenia	4/16/2021	10.1056/nejme2106315	332
1877	Multiorgan and Renal Tropism of SARS-CoV-2	8/6/2020	10.1056/nejmc2011400	1264
1838	Expression of Concern: Mehra MR et al. Cardiovascular Disease, Drug Therapy, and Mortality in COVID-19. N Engl J Med. DOI: 10.1056/NEJMoa2007621.	6/18/2020	10.1056/nejme2020822	26
1835	Universal Masking in the COVID-19 Era	7/9/2020	10.1056/nejmc2020836	16
1826	Phase 1–2 Trial of a SARS-CoV-2 Recombinant Spike Protein Nanoparticle Vaccine	12/10/2020	10.1056/nejmoa2026920	819
1822	Effectiveness of mRNA COVID-19 Vaccine among U.S. Health Care Personnel	12/16/2021	10.1056/nejmoa2106599	135
1809	Evidence of SARS-CoV-2 Infection in Returning Travelers from Wuhan, China	3/26/2020	10.1056/nejmc2001899	479
1801	SARS-CoV-2 Variants in Patients with Immunosuppression	8/5/2021	10.1056/nejmsb2104756	189
1785	COVID-19 and the Stiff Upper Lip—The Pandemic Response in the United Kingdom	4/16/2020	10.1056/nejmp2005755	122
1766	COVID-19 Vaccine Protection among Children and Adolescents in Qatar	11/2/2022	10.1056/nejmoa2210058	0
1743	Severe COVID-19	12/17/2020	10.1056/nejmcp2009575	880
1742	New SARS-CoV-2 Variants—Clinical, Public Health, and Vaccine Implications	5/13/2021	10.1056/nejmc2100362	485
1733	Facing COVID-19 in Italy—Ethics, Logistics, and Therapeutics on the Epidemic's Front Line	5/14/2020	10.1056/nejmp2005492	653
1732	Intramuscular AZD7442 (Tixagevimab–Cilgavimab) for Prevention of COVID-19	6/9/2022	10.1056/nejmoa2116620	120
1690	BNT162b2 Vaccine Effectiveness against Omicron in Children 5 to 11 Years of Age	7/21/2022	10.1056/nejmoa2205011	15
1675	Effectiveness of BNT162b2 vaccine against SARS-CoV-2 infection and severe COVID-19 in children aged 5–11 years in Italy: a retrospective analysis of January–April, 2022	7/1/2022	10.1016/s0140-6736(22)01185-0	16
1667	Renin–Angiotensin–Aldosterone System Inhibitors in Patients with COVID-19	4/23/2020	10.1056/NEJMsr2005760	1605
1617	Effectiveness of the BNT162b2 Vaccine after Recovery from COVID-19	3/31/2022	10.1056/nejmoa2119497	42
1616	REGEN-COV Antibody Combination and Outcomes in Outpatients with COVID-19	12/2/2021	10.1056/nejmoa2108163	288
1596	Maternal Vaccination and Risk of Hospitalization for COVID-19 among Infants	7/14/2022	10.1056/nejmoa2204399	21
1583	The Untold Toll—The Pandemic's Effects on Patients without COVID-19	6/11/2020	10.1056/nejmms2009984	497
1577	Early Convalescent Plasma for High-Risk Outpatients with COVID-19	11/18/2021	10.1056/nejmoa2103784	106
1554	Coagulopathy and Antiphospholipid Antibodies in Patients with COVID-19	4/23/2020	10.1056/nejmc2007575	1671
1534	Evaluation of mRNA-1273 COVID-19 Vaccine in Children 6 to 11 Years of Age	5/26/2022	10.1056/nejmoa2203315	26
1523	Maintaining Safety with SARS-CoV-2 Vaccines	2/18/2021	10.1056/nejmra2035343	279
1518	Challenges in Inferring Intrinsic Severity of the SARS-CoV-2 Omicron Variant	2/17/2022	10.1056/nejmp2119682	73
1507	Detection of COVID-19 in Children in Early January 2020 in Wuhan, China	4/2/2020	10.1056/nejmc2003717	521
1493	Effect of mRNA Vaccine Boosters against SARS-CoV-2 Omicron Infection in Qatar	5/12/2022	10.1056/nejmoa2200797	125
1486	Preliminary Findings of mRNA COVID-19 Vaccine Safety in Pregnant Persons	10/14/2021	10.1056/nejmx210016	4
1470	Epidemiology of COVID-19 in a Long-Term Care Facility in King County, Washington	5/21/2020	10.1056/nejmoa2005412	1044
1468	Multisystem Inflammatory Syndrome in Children in New York State	7/23/2020	10.1056/nejmoa2021756	840
1468	Early Treatment for COVID-19 with SARS-CoV-2 Neutralizing Antibody Sotrovimab	11/18/2021	10.1056/nejmoa2107934	479
1459	Losing Contact—COVID-19, Telemedicine, and the Patient–Provider Relationship	9/1/2022	10.1056/nejmp2206471	0
1430	Rapid Diagnostic Testing for SARS-CoV-2	1/20/2022	10.1056/nejmcp2117115	49
1420	Susceptibility of Circulating SARS-CoV-2 Variants to Neutralization	4/6/2021	10.1056/nejmc2103022	130
1413	Critical Supply Shortages—The Need for Ventilators and Personal Protective Equipment during the COVID-19 Pandemic	4/30/2020	10.1056/nejmp2006141	1384
1410	Neutralization of the SARS-CoV-2 Omicron BA.1 and BA.2 Variants	4/21/2022	10.1056/nejmc2201849	178
1407	Beyond the First Dose—COVID-19 Vaccine Follow-through and Continued Protective Measures	7/8/2021	10.1056/nejmp2104527	15
1399	The COVID-19 Pandemic and the Incidence of Acute Myocardial Infarction	8/13/2020	10.1056/nejmc2015630	479
1359	Drug Evaluation during the COVID-19 Pandemic	6/11/2020	10.1056/nejmp2009457	126
1348	Duration of Protection against Mild and Severe Disease by COVID-19 Vaccines	1/27/2022	10.1056/nejmoa2115481	245
1340	IL-1RA Antibodies in Myocarditis after SARS-CoV-2 Vaccination	10/20/2022	10.1056/nejmc2205667	1
1295	Efficacy of Antibodies and Antiviral Drugs against COVID-19 Omicron Variant	3/10/2022	10.1056/nejmc2119407	172
1292	Interleukin-6 Receptor Antagonists in Critically Ill Patients with COVID-19	4/22/2021	10.1056/nejmoa2100433	1017
1290	Myocarditis after COVID-19 mRNA Vaccination	9/30/2021	10.1056/nejmc2109975	122
1269	Hydroxychloroquine for the Prevention of COVID-19—Searching for Evidence	8/6/2020	10.1056/nejme2020388	76
1262	Audio Interview: COVID-19 Vaccines and Pregnancy-A Conversation with CDC Director Rochelle Walensky	4/22/2021	10.1056/nejme2106836	0
1257	Efficacy of NVX-CoV2373 COVID-19 Vaccine against the B.1.351 Variant	5/20/2021	10.1056/nejmoa2103055	417
1243	A Pandemic within a Pandemic—Intimate Partner Violence during COVID-19	12/10/2020	10.1056/nejmp2024046	215
1219	Homologous and Heterologous COVID-19 Booster Vaccinations	3/17/2022	10.1056/nejmoa2116414	193
1215	Neutralization of the SARS-CoV-2 Mu Variant by Convalescent and Vaccine Serum	12/16/2021	10.1056/nejmc2114706	146
1205	Effectiveness of COVID-19 Vaccines over a 9-Month Period in North Carolina	3/10/2022	10.1056/nejmoa2117128	110
1187	"Is It Safe for Me to Go to Work?" Risk Stratification for Workers during the COVID-19 Pandemic	7/30/2020	10.1056/nejmp2013413	60
1138	Virtually Perfect? Telemedicine for COVID-19	4/30/2020	10.1056/nejmp2003539	1942
1132	Hospitalization and Mortality among Black Patients and White Patients with COVID-19	6/25/2020	10.1056/nejmsa2011686	1242
1127	Infection and Vaccine-Induced Neutralizing-Antibody Responses to the SARS-CoV-2 B.1.617 Variants	8/12/2021	10.1056/nejmc2107799	225
1105	An Outbreak of COVID-19 on an Aircraft Carrier	12/17/2020	10.1056/nejmoa2019375	95
1096	Time to Stop Using Ineffective COVID-19 Drugs	8/18/2022	10.1056/nejme2209017	3
1085	SARS-CoV-2 Antibody Response in Persons with Past Natural Infection	7/1/2021	10.1056/nejmc2103825	103
1083	Efficacy of Tocilizumab in Patients Hospitalized with COVID-19	12/10/2020	10.1056/nejmoa2028836	962
1077	Therapeutic Anticoagulation with Heparin in Noncritically Ill Patients with COVID-19	8/26/2021	10.1056/nejmoa2105911	414
1070	Detection of SARS-CoV-2 with SHERLOCK One-Pot Testing	10/8/2020	10.1056/nejmc2026172	352
1052	Duration of Culturable SARS-CoV-2 in Hospitalized Patients with COVID-19	2/18/2021	10.1056/nejmc2027040	127
1048	Mild or Moderate COVID-19	10/29/2020	10.1056/nejmcp2009249	864
1046	Remdesivir for 5 or 10 Days in Patients with Severe COVID-19	11/5/2020	10.1056/nejmoa2015301	982
1044	Spread of a Variant SARS-CoV-2 in Long-Term Care Facilities in England	4/29/2021	10.1056/nejmc2035906	21
1044	Lung Transplantation for COVID-19-Related Respiratory Failure in the United States	3/24/2022	10.1056/nejmc2117024	31
1039	Mental Health and the COVID-19 Pandemic	8/6/2020	10.1056/nejmp2008017	2544
1037	Neutralizing Response against Variants after SARS-CoV-2 Infection and One Dose of BNT162b2	6/24/2021	10.1056/nejmc2104036	62
1028	Adverse Effects after BNT162b2 Vaccine and SARS-CoV-2 Infection, According to Age and Sex	12/9/2021	10.1056/nejmc2115045	19
1025	Natural History of Asymptomatic SARS-CoV-2 Infection	8/27/2020	10.1056/nejmc2013020	228
1025	REGN-COV2, a Neutralizing Antibody Cocktail, in Outpatients with COVID-19	1/21/2021	10.1056/nejmoa2035002	1204
1025	Neutralization of SARS-CoV-2 Variants B.1.429 and B.1.351	4/7/2021	10.1056/nejmc2103740	172
1018	Tocilizumab in Hospitalized Patients with Severe COVID-19 Pneumonia	4/22/2021	10.1056/nejmoa2028700	554
1009	Phase 3 Safety and Efficacy of AZD1222 (ChAdOx1 nCoV-19) COVID-19 Vaccine	12/16/2021	10.1056/nejmoa2105290	234
1007	The COVID-19 Infodemic—Applying the Epidemiologic Model to Counter Misinformation	8/19/2021	10.1056/nejmp2103798	43
979	Shedding of Viable SARS-CoV-2 after Immunosuppressive Therapy for Cancer	12/24/2020	10.1056/nejmc2031670	251
978	Out-of-Hospital Cardiac Arrest during the COVID-19 Outbreak in Italy	7/30/2020	10.1056/nejmc2010418	461
975	Comparative Effectiveness of BNT162b2 and mRNA-1273 Vaccines in U.S. Veterans	1/13/2022	10.1056/nejmoa2115463	98
965	Droplets and Aerosols in the Transmission of SARS-CoV-2	5/21/2020	10.1056/nejmc2009324	235
945	History in a Crisis—Lessons for COVID-19	4/30/2020	10.1056/nejmp2004361	222
939	Pulse Oximetry for Monitoring Patients with COVID-19 at Home—A Pragmatic, Randomized Trial	5/12/2022	10.1056/nejmc2201541	1
938	Resistance Mutations in SARS-CoV-2 Delta Variant after Sotrovimab Use	4/14/2022	10.1056/nejmc2120219	61
934	The Future of SARS-CoV-2 Vaccination—Lessons from Influenza	11/11/2021	10.1056/nejmp2113403	35
916	The COVID-19 Vaccine-Development Multiverse	11/12/2020	10.1056/nejme2025111	97
909	Differential Kinetics of Immune Responses Elicited by COVID-19 Vaccines	11/18/2021	10.1056/nejmc2115596	140
898	COVID-19—A Reminder to Reason	7/16/2020	10.1056/nejmp2009405	89
891	Nursing Home Staff Vaccination and COVID-19 Outcomes	1/27/2022	10.1056/nejmc2115674	17
885	Third BNT162b2 Vaccination Neutralization of SARS-CoV-2 Omicron Infection	2/3/2022	10.1056/nejmc2119358	263
882	Clinical Characteristics of COVID-19 in New York City	6/11/2020	10.1056/nejmc2010419	1681
875	Emergence of a Highly Fit SARS-CoV-2 Variant	12/31/2020	10.1056/nejmcibr2032888	130
866	Vaccinating Children against COVID-19—The Lessons of Measles	2/18/2021	10.1056/nejmp2034765	48
850	Efficacy of Antiviral Agents against the SARS-CoV-2 Omicron Subvariant BA.2	4/14/2022	10.1056/nejmc2201933	133
829	Defining the Epidemiology of COVID-19—Studies Needed	3/26/2020	10.1056/nejmp2002125	907
828	A Neutralizing Monoclonal Antibody for Hospitalized Patients with COVID-19	3/11/2021	10.1056/nejmoa2033130	291
828	Accelerating Development of SARS-CoV-2 Vaccines—The Role for Controlled Human Infection Models	9/3/2020	10.1056/nejmp2020076	63
819	Bamlanivimab plus Etesevimab in Mild or Moderate COVID-19	10/7/2021	10.1056/nejmoa2102685	333
808	Safety and Efficacy of a Third Dose of BNT162b2 COVID-19 Vaccine	5/19/2022	10.1056/nejmoa2200674	62
796	Rapid Scaling Up of COVID-19 Diagnostic Testing in the United States—The NIH RADx Initiative	9/10/2020	10.1056/nejmsr2022263	151
795	Convalescent Plasma Antibody Levels and the Risk of Death from COVID-19	3/18/2021	10.1056/nejmoa2031893	358
777	Racial Health Disparities and COVID-19—Caution and Context	7/16/2020	10.1056/nejmp2012910	494
775	Therapeutic Anticoagulation with Heparin in Critically Ill Patients with COVID-19	8/26/2021	10.1056/nejmoa2103417	451
771	Incident SARS-CoV-2 Infection among mRNA-Vaccinated and Unvaccinated Nursing Home Residents	7/29/2021	10.1056/nejmc2104849	56
766	COVID-19—The Search for Effective Therapy	5/7/2020	10.1056/nejme2005477	241
758	Renin–Angiotensin–Aldosterone System Inhibitors and Risk of COVID-19	6/18/2020	10.1056/nejmoa2008975	874
745	Early Outpatient Treatment for COVID-19 with Convalescent Plasma	5/5/2022	10.1056/nejmoa2119657	65
734	Sparing of Severe COVID-19 in Vaccinated Adolescents	2/24/2022	10.1056/nejme2118471	2
729	Collateral Effect of COVID-19 on Stroke Evaluation in the United States	7/23/2020	10.1056/nejmc2014816	360
724	SARS-CoV-2 Variants and Vaccines	7/8/2021	10.1056/nejmsr2105280	239
723	Omicron SARS-CoV-2 Neutralization from Inactivated and ZF2001 Vaccines	7/21/2022	10.1056/nejmc2206900	11
720	Beyond Politics—Promoting COVID-19 Vaccination in the United States	2/18/2021	10.1056/nejmms2033790	108
719	Durability of Booster mRNA Vaccine against SARS-CoV-2 BA.2.12.1, BA.4, and BA.5 Subvariants	10/6/2022	10.1056/nejmc2210546	5
714	Delayed Second Dose versus Standard Regimen for COVID-19 Vaccination	3/4/2021	10.1056/nejmclde2101987	56
710	COVID-19 and Disparities in Nutrition and Obesity	9/10/2020	10.1056/nejmp2021264	148
706	Not Dying Alone—Modern Compassionate Care in the COVID-19 Pandemic	6/11/2020	10.1056/nejmp2007781	153
704	Feeding Low-Income Children during the COVID-19 Pandemic	4/30/2020	10.1056/nejmp2005638	208
696	SARS-CoV-2 Vaccination—An Ounce (Actually, Much Less) of Prevention	12/31/2020	10.1056/nejme2034717	46
696	Tocilizumab in Patients Hospitalized with COVID-19 Pneumonia	1/7/2021	10.1056/nejmoa2030340	827
695	COVID-19 Vaccine Effectiveness in New York State	1/13/2022	10.1056/nejmoa2116063	128
686	Universal Masking Policies in Schools and Mitigating the Inequitable Costs of COVID-19	11/9/2022	10.1056/nejme2213556	0
680	COVID-19 Vaccines—Immunity, Variants, Boosters	9/15/2022	10.1056/nejmra2206573	15
679	Tofacitinib in Patients Hospitalized with COVID-19 Pneumonia	7/29/2021	10.1056/nejmoa2101643	211
674	SARS-CoV-2 Omicron Variant Neutralization in Serum from Vaccinated and Convalescent Persons	2/17/2022	10.1056/nejmc2119236	249
669	Swabs Collected by Patients or Health Care Workers for SARS-CoV-2 Testing	7/30/2020	10.1056/nejmc2016321	210
668	Protection with a Third Dose of mRNA Vaccine against SARS-CoV-2 Variants in Frontline Workers	5/12/2022	10.1056/nejmc2201821	21
668	Vaccination plus Decarceration—Stopping COVID-19 in Jails and Prisons	4/29/2021	10.1056/nejmp2100609	42
667	Wrong but Useful—What COVID-19 Epidemiologic Models Can and Cannot Tell Us	7/23/2020	10.1056/nejmp2016822	301
649	Breakthrough SARS-CoV-2 Infections in Prison after Vaccination	9/9/2021	10.1056/nejmc2108479	23
643	mRNA COVID-19 Vaccines in Pregnant Women	10/14/2021	10.1056/nejmx210017	0
634	"When Will We Have a Vaccine?"—Understanding Questions and Answers about COVID-19 Vaccination	12/3/2020	10.1056/nejmp2025331	57
631	The Importance of Context in COVID-19 Vaccine Safety	9/16/2021	10.1056/nejme2112543	9
630	BNT162b2 mRNA COVID-19 Vaccine Effectiveness among Health Care Workers	5/6/2021	10.1056/nejmc2101951	114
625	ST-Segment Elevation in Patients with COVID-19—A Case Series	6/18/2020	10.1056/nejmc2009020	573
624	Anti-Spike Mucosal IgA Protection against SARS-CoV-2 Omicron Infection	10/6/2022	10.1056/nejmc2209651	3
613	Adjunct Immune Globulin for Vaccine-Induced Immune Thrombotic Thrombocytopenia	8/19/2021	10.1056/nejmoa2107051	117
607	Neuropathological Features of COVID-19	9/3/2020	10.1056/nejmc2019373	545
590	Association between COVID-19 Vaccination and Influenza Vaccination Rates	6/30/2022	10.1056/nejmc2204560	6
590	Hydroxychloroquine in Hospitalized Patients with COVID-19	3/4/2021	10.1056/nejmc2035374	6
584	Neutralization Escape by SARS-CoV-2 Omicron Subvariant BA.4.6	10/19/2022	10.1056/nejmc2212117	1
577	COVID-19 Vaccine Injuries—Preventing Inequities in Compensation	3/11/2021	10.1056/nejmp2034438	13
567	Efficacy and Safety of a Recombinant Plant-Based Adjuvanted COVID-19 Vaccine	6/2/2022	10.1056/nejmoa2201300	33
565	Compassionate Use of Remdesivir in COVID-19	6/18/2020	10.1056/nejmc2015312	89
560	Does the World Still Need New COVID-19 Vaccines?	6/2/2022	10.1056/nejme2204695	18
555	Children with COVID-19 in Pediatric Emergency Departments in Italy	7/9/2020	10.1056/nejmc2007617	492
549	Immune Imprinting and Protection against Repeat Reinfection with SARS-CoV-2	11/3/2022	10.1056/nejmc2211055	3
548	Adolescents, Parents, and COVID-19 Vaccination—Who Should Decide?	1/13/2022	10.1056/nejmp2116771	7
542	Cross-Reactive Neutralizing Antibody Responses Elicited by SARS-CoV-2 501Y.V2 (B.1.351)	6/3/2021	10.1056/nejmc2104192	93
541	Renin–Angiotensin–Aldosterone System Blockers and the Risk of COVID-19	6/18/2020	10.1056/nejmoa2006923	885
524	No-Fault Compensation for Vaccine Injury—The Other Side of Equitable Access to COVID-19 Vaccines	12/3/2020	10.1056/nejmp2030600	26
523	The Urgency of Care during the COVID-19 Pandemic—Learning as We Go	6/18/2020	10.1056/nejme2015903	36
523	Loss of Anti-SARS-CoV-2 Antibodies in Mild COVID-19	10/22/2020	10.1056/nejmc2027051	68
517	Placebo-Controlled Trials of COVID-19 Vaccines—Why We Still Need Them	1/14/2021	10.1056/nejmp2033538	58
506	Rebound of SARS-CoV-2 Infection after Nirmatrelvir–Ritonavir Treatment	9/15/2022	10.1056/nejmc2206449	4
489	The Missing Piece—SARS-CoV-2 Testing and School Reopening	12/3/2020	10.1056/nejmp2028209	23
488	Trustworthiness before Trust—COVID-19 Vaccine Trials and the Black Community	11/26/2020	10.1056/nejmp2030033	134
486	COVID-19 Breakthrough Infections in Vaccinated Health Care Workers	10/21/2021	10.1056/nejmc2113497	26
485	COVID-19 Vaccine Effectiveness and the Test-Negative Design	10/7/2021	10.1056/nejme2113151	70
481	Thinking Globally, Acting Locally—The U.S. Response to COVID-19	5/28/2020	10.1056/nejmp2006740	209
474	Science, Competing Values, and Trade-offs in Public Health—The Example of COVID-19 and Masking	9/8/2022	10.1056/nejmp2207670	1
465	Population Immunity and COVID-19 Severity with Omicron Variant in South Africa	4/7/2022	10.1056/nejmoa2119658	144
464	Plasma Neutralization of the SARS-CoV-2 Omicron Variant	2/10/2022	10.1056/nejmc2119641	281
458	COVID-19—Implications for the Health Care System	10/8/2020	10.1056/nejmsb2021088	355
456	An Uncertain Public—Encouraging Acceptance of COVID-19 Vaccines	4/22/2021	10.1056/nejmp2100351	57
452	Flattening the Curve for Incarcerated Populations—COVID-19 in Jails and Prisons	5/28/2020	10.1056/nejmp2005687	196
446	Open Schools, COVID-19, and Child and Teacher Morbidity in Sweden	4/29/2021	10.1056/nejmc2101280	7
441	Immunogenicity and Reactogenicity of Vaccine Boosters after Ad26.COV2.S Priming	3/10/2022	10.1056/nejmoa2116747	48
433	Facial Masking for COVID-19	11/19/2020	10.1056/nejmc2030886	16
431	COVID-19 and Health Care's Digital Revolution	6/4/2020	10.1056/nejmp2005835	594
429	Cognitive Deficits in Long COVID-19	11/10/2022	10.1056/nejmcibr2210069	0
423	Between Scylla and Charybdis—Oncologic Decision Making in the Time of COVID-19	6/11/2020	10.1056/nejmp2006588	60
420	The FDA's Experience with COVID-19 Antibody Tests	2/18/2021	10.1056/nejmp2033687	18
412	Undocumented U.S. Immigrants and COVID-19	5/21/2020	10.1056/nejmp2005953	172
411	The Climate Crisis and COVID-19—A Major Threat to the Pandemic Response	9/10/2020	10.1056/nejmp2022011	50
411	Inhibitors of the Renin–Angiotensin–Aldosterone System and COVID-19	6/18/2020	10.1056/nejme2012924	102
411	Waiting for Certainty on COVID-19 Antibody Tests—At What Cost?	8/6/2020	10.1056/nejmp2017739	76
406	Protective Effect of Previous SARS-CoV-2 Infection against Omicron BA.4 and BA.5 Subvariants	10/27/2022	10.1056/nejmc2209306	6
398	Late-Onset Neonatal Sepsis in a Patient with COVID-19	5/7/2020	10.1056/nejmc2010614	90
395	Death from COVID-19 of 23 Health Care Workers in China	6/4/2020	10.1056/nejmc2005696	246
382	Public Health Law after COVID-19	9/23/2021	10.1056/nejmp2112193	5
380	Antibody Responses after a Single Dose of SARS-CoV-2 mRNA Vaccine	5/20/2021	10.1056/nejmc2102051	117
379	Supporting Clinicians during COVID-19 and Beyond—Learning from Past Failures and Envisioning New Strategies	12/31/2020	10.1056/nejmp2024834	37
370	COVID-19 and Health Equity—Time to Think Big	9/17/2020	10.1056/nejmp2021209	62
370	Neutralization of SARS-CoV-2 Omicron BA.2.75 after mRNA-1273 Vaccination	9/29/2022	10.1056/nejmc2210648	2
367	Trained Innate Immunity, Epigenetics, and COVID-19	9/10/2020	10.1056/nejmcibr2011679	104
364	Neutralization of the SARS-CoV-2 Omicron BA.4/5 and BA.2.12.1 Subvariants	6/30/2022	10.1056/nejmc2206725	71
361	BNT162b2-Elicited Neutralization against New SARS-CoV-2 Spike Variants	7/29/2021	10.1056/nejmc2106083	80
350	COVID-19 Vaccination in American Indians and Alaska Natives—Lessons from Effective Community Responses	12/23/2021	10.1056/nejmp2113296	13
345	Reduced Rate of Hospital Admissions for ACS during COVID-19 Outbreak in Northern Italy	7/2/2020	10.1056/nejmc2009166	735
341	Long-Term Care Policy after COVID-19—Solving the Nursing Home Crisis	9/3/2020	10.1056/nejmp2014811	81
339	COVID-19 Molecular Diagnostic Testing—Lessons Learned	10/22/2020	10.1056/nejmp2023830	27
336	On Preliminary Findings of mRNA COVID-19 Vaccine Safety in Pregnant Persons	10/14/2021	10.1056/nejmc2113516	4
333	COVID-19 Vaccination during Pregnancy—Two for the Price of One	7/14/2022	10.1056/nejme2206730	1
331	Delayed Large Local Reactions to mRNA COVID-19 Vaccines in Blacks, Indigenous Persons, and People of Color	8/12/2021	10.1056/nejmc2108620	12
317	Incentives for Immunity—Strategies for Increasing COVID-19 Vaccine Uptake	7/1/2021	10.1056/nejmp2107719	43
315	COVID-19 in Immune-Mediated Inflammatory Diseases—Case Series from New York	7/2/2020	10.1056/nejmc2009567	365
309	Immune Thrombocytopenic Purpura in a Patient with COVID-19	4/30/2020	10.1056/nejmc2010472	230
309	COVID-19 and Kidney Transplantation	6/18/2020	10.1056/nejmc2011117	587
296	COVID-19—The Law and Limits of Quarantine	4/9/2020	10.1056/nejmp2004211	279
293	Missing the Point—How Primary Care Can Overcome COVID-19 Vaccine "Hesitancy"	6/24/2021	10.1056/nejmp2106137	32
293	Early Detection of COVID-19 through a Citywide Pandemic Surveillance Platform	7/9/2020	10.1056/nejmc2008646	86
287	Mass-Vaccination Sites—An Essential Innovation to Curb the COVID-19 Pandemic	5/6/2021	10.1056/nejmp2102535	46
280	Up Is Down—Pharmaceutical Industry Caution vs. Federal Acceleration of COVID-19 Vaccine Approval	10/29/2020	10.1056/nejmp2029479	10
279	Personal Protective Equipment and COVID-19	6/25/2020	10.1056/nejmvcm2014809	62
278	(A Little) Clarity on Convalescent Plasma for COVID-19	2/18/2021	10.1056/nejme2035678	73
278	Africa in the Path of COVID-19	7/16/2020	10.1056/nejmp2008193	147
269	Effects of BNT162b2 COVID-19 Vaccine Booster in Long-Term Care Facilities in Israel	1/27/2022	10.1056/nejmc2117385	20
266	Effectiveness of an Inactivated SARS-CoV-2 Vaccine	9/2/2021	10.1056/nejme2111165	29
266	Lessons We've Learned—COVID-19 and the Undocumented Latinx Community	1/7/2021	10.1056/nejmp2024897	54
266	Abortion during the COVID-19 Pandemic—Ensuring Access to an Essential Health Service	5/7/2020	10.1056/nejmp2008006	73
264	Receipt of mRNA Vaccine against COVID-19 and Myocarditis	12/2/2021	10.1056/nejme2116493	12
264	Clinical Characteristics of Pregnant Women with COVID-19 in Wuhan, China	6/18/2020	10.1056/nejmc2009226	400
259	Structural Racism, Social Risk Factors, and COVID-19—A Dangerous Convergence for Black Americans	9/17/2020	10.1056/nejmp2023616	332
257	Ebola Response Priorities in the Time of COVID-19	9/24/2020	10.1056/nejmp2025512	8
255	Efficacy of Natural Immunity against SARS-CoV-2 Reinfection with the Beta Variant	12/30/2021	10.1056/nejmc2110300	48
254	Final Analysis of Efficacy and Safety of Single-Dose Ad26.COV2.S	3/3/2022	10.1056/nejmoa2117608	57
253	Clinical Characteristics of COVID-19 in China	5/7/2020	10.1056/nejmc2005203	149
247	Disease Control, Civil Liberties, and Mass Testing—Calibrating Restrictions during the COVID-19 Pandemic	7/9/2020	10.1056/nejmp2007637	119
245	Effectiveness of COVID-19 Vaccines against the B.1.617.2 (Delta) Variant	12/16/2021	10.1056/nejmc2113090	17
244	Lupus Anticoagulant and Abnormal Coagulation Tests in Patients with COVID-19	7/16/2020	10.1056/nejmc2013656	375
238	Acute Cor Pulmonale in Critically Ill Patients with COVID-19	5/21/2020	10.1056/nejmc2010459	101
237	Audio Interview: A COVID-19 Conversation with Anthony Fauci	1/28/2021	10.1056/nejme2101618	4
237	A Locally Transmitted Case of SARS-CoV-2 Infection in Taiwan	3/12/2020	10.1056/nejmc2001573	109
232	Interleukin-6 Receptor Inhibition in COVID-19—Cooling the Inflammatory Soup	4/22/2021	10.1056/nejme2103108	87
232	Audio Interview: Making Decisions about COVID-19 Testing and Treatment for Your Patients	3/12/2020	10.1056/nejme2004856	9
224	Hydroxychloroquine with or without Azithromycin in Mild-to-Moderate COVID-19	11/19/2020	10.1056/nejmx200021	43
223	COVID-19 and Immunity in Aging Populations—A New Research Agenda	8/27/2020	10.1056/nejmp2006761	145
219	Interplay between Emerging SARS-CoV-2 Variants and Pandemic Control	5/20/2021	10.1056/nejme2103931	17
218	Community Health Centers and COVID-19—Time for Congress to Act	8/20/2020	10.1056/nejmp2020576	16
215	Addressing Vaccine Inequity—COVID-19 Vaccines as a Global Public Good	3/24/2022	10.1056/nejme2202547	25
215	VITT and Second Doses of COVID-19 Vaccine	1/6/2022	10.1056/nejmc2118507	26
214	Effectiveness of Homologous and Heterologous COVID-19 Boosters against Omicron	6/23/2022	10.1056/nejmc2203165	14
213	Effectiveness of the mRNA-1273 Vaccine during a SARS-CoV-2 Delta Outbreak in a Prison	12/9/2021	10.1056/nejmc2114089	24
213	Neutralization of the SARS-CoV-2 Deltacron and BA.3 Variants	6/16/2022	10.1056/nejmc2205019	11
212	Contact Tracing for COVID-19—A Digital Inoculation against Future Pandemics	8/5/2021	10.1056/nejmp2102256	20
211	Surviving COVID-19 with Heparin?	8/26/2021	10.1056/nejme2111151	42
203	Caring for the Caregivers—COVID-19 Vaccination for Essential Members of the Health Care Team	3/4/2021	10.1056/nejmpv2101339	8
202	Audio Interview: COVID-19—The Outlook in Europe	1/6/2022	10.1056/nejme2200149	0
201	Emergency Intubation in COVID-19	2/18/2021	10.1056/nejmvcm2007198	6
199	Protection against Omicron from Vaccination and Previous Infection in a Prison System	11/10/2022	10.1056/nejmoa2207082	0
195	Delaying Pregnancy during a Public Health Crisis—Examining Public Health Recommendations for COVID-19 and Beyond	11/26/2020	10.1056/nejmp2027940	8
195	COVID-19 in South Korea—Challenges of Subclinical Manifestations	5/7/2020	10.1056/nejmc2001801	100
191	Bridging the Gap at Warp Speed—Delivering Options for Preventing and Treating COVID-19	11/12/2020	10.1056/nejmp2028535	13
190	Monoclonal Antibodies to Disrupt Progression of Early COVID-19 Infection	1/21/2021	10.1056/nejme2034495	66
187	A Comprehensive COVID-19 Response—The Need for Economic Evaluation	6/30/2022	10.1056/nejmp2202828	3
177	SARS-CoV-2 in the U.S. Military—Lessons for Civil Society	12/17/2020	10.1056/nejme2032179	12
175	Stability and Viability of SARS-CoV-2	5/14/2020	10.1056/nejmc2007942	37
171	Risk Factors for SARS-CoV-2 in a Statewide Correctional System	12/17/2020	10.1056/nejmc2029354	16
166	An Uncomplicated Delivery in a Patient with COVID-19 in the United States	4/16/2020	10.1056/nejmc2007605	90
164	Evaluating and Deploying COVID-19 Vaccines—The Importance of Transparency, Scientific Integrity, and Public Trust	10/29/2020	10.1056/nejmp2026393	51
162	Vaccine Breakthrough Infections with SARS-CoV-2 Variants	7/8/2021	10.1056/nejmc2107808	31
153	COVID-19 and the Need for Health Care Reform	6/25/2020	10.1056/nejmp2000821	81
153	COVID-19 Vaccine Acceptance in California State Prisons	7/22/2021	10.1056/nejmc2105282	25
151	Evaluation of Acute Adverse Events after COVID-19 Vaccination during Pregnancy	7/14/2022	10.1056/nejmc2205276	2
150	Uncomfortable Truths—What COVID-19 Has Revealed about Chronic-Disease Care in America	10/28/2021	10.1056/nejmp2112063	12
149	Who Goes First? Government Leaders and Prioritization of SARS-CoV-2 Vaccines	2/4/2021	10.1056/nejmpv2036128	5
149	Community Health Workers and COVID-19—Addressing Social Determinants of Health in Times of Crisis and Beyond	11/5/2020	10.1056/nejmp2022641	77
146	Efficacy and Safety of the RBD-Dimer-Based COVID-19 Vaccine ZF2001 in Adults	6/2/2022	10.1056/nejmoa2202261	30
146	Hydroxychloroquine as Postexposure Prophylaxis for COVID-19	9/10/2020	10.1056/nejmc2023617	18
144	Not a Perfect Storm—COVID-19 and the Importance of Language	4/16/2020	10.1056/nejmp2005032	13
142	Anticoagulation in Hospitalized Patients with COVID-19	10/22/2020	10.1056/nejmclde2028217	40
138	Business Not as Usual—COVID-19 Vaccination in Persons with Substance Use Disorders	1/14/2021	10.1056/nejmpv2035709	19
137	Protection Associated with Previous SARS-CoV-2 Infection in Nicaragua	8/11/2022	10.1056/nejmc2203985	2
136	Third Time's a Charm—COVID-19 Vaccine Hope for Solid-Organ Transplant Recipients	9/23/2021	10.1056/nejme2112866	10
135	Prevention of COVID-19 with the BNT162b2 and mRNA-1273 Vaccines	11/4/2021	10.1056/nejmc2113575	14
134	COVID-19 mRNA Vaccines—Six of One, Half a Dozen of the Other	1/13/2022	10.1056/nejme2117446	11
132	Mining a GWAS of Severe COVID-19	12/24/2020	10.1056/nejmc2025747	8
132	Added Benefit of COVID-19 Vaccination after Previous Infection	3/31/2022	10.1056/nejme2201380	3
128	Molnupiravir—A Step toward Orally Bioavailable Therapies for COVID-19	2/10/2022	10.1056/nejme2117814	29
128	More on BNT162b2 COVID-19 Vaccine in Children 5 to 11 Years of Age	3/24/2022	10.1056/nejmc2201556	4
122	Nirmatrelvir–Ritonavir and Viral Load Rebound in COVID-19	9/15/2022	10.1056/nejmc2205944	4
121	SARS-CoV-2 Infection in Patients with a History of VITT	7/7/2022	10.1056/nejmc2206601	1
120	BNT162b2 mRNA COVID-19 Vaccine in a Nationwide Mass Vaccination Setting	5/20/2021	10.1056/nejmc2104281	23
119	Cancer Management in India during COVID-19	5/14/2020	10.1056/nejmc2011595	99
118	Audio Interview: India's COVID-19 Crisis	5/6/2021	10.1056/nejme2107728	10
118	COVID-19 Crisis Triage—Optimizing Health Outcomes and Disability Rights	7/30/2020	10.1056/nejmp2008300	56
115	SARS-CoV-2 Infection among Travelers Returning from Wuhan, China	4/9/2020	10.1056/nejmc2003100	97
111	Monoclonal Antibodies with Extended Half-Life to Prevent COVID-19	6/9/2022	10.1056/nejme2205563	0
109	Preparing for the Future—Nanobodies for COVID-19?	4/22/2021	10.1056/nejmcibr2101205	20
108	Case 40-2020: A 24-Year-Old Man with Headache and COVID-19	12/24/2020	10.1056/nejmcpc2027083	15
107	SARS-CoV-2 Human Challenge Studies—Establishing the Model during an Evolving Pandemic	9/9/2021	10.1056/nejmp2106970	26
104	COVID-19, Ebola, and HIV—Leveraging Lessons to Maximize Impact	11/5/2020	10.1056/nejmp2022269	15
99	Audio Interview: Emerging Tools in the Fight against COVID-19	4/9/2020	10.1056/nejme2009651	3
99	SARS-CoV-2 Evolution and Immune Escape in Immunocompromised Patients	6/23/2022	10.1056/nejmc2202861	9
98	Improving Clinical Trial Enrollment—In the COVID-19 Era and Beyond	10/8/2020	10.1056/nejmp2019989	39
98	Not Ready for the End Game—Why Ending Federal COVID-19 Emergency Declarations Will Harm Access to Care	4/21/2022	10.1056/nejmp2203468	2
92	The Stress of Bayesian Medicine—Uncomfortable Uncertainty in the Face of COVID-19	1/7/2021	10.1056/nejmp2018857	8
91	Public Health Decision Making during COVID-19—Fulfilling the CDC Pledge to the American People	9/3/2020	10.1056/nejmp2026045	10
91	COVID-19, Angiogenesis, and ARDS Endotypes	7/9/2020	10.1056/nejme2018629	70
91	A New Vaccine to Battle COVID-19	2/4/2021	10.1056/nejme2035557	42
89	CPR in the COVID-19 Era—An Ethical Framework	7/9/2020	10.1056/nejmp2010758	54
85	Omicron BA.1/1.1 SARS-CoV-2 Infection among Vaccinated Canadian Adults	6/16/2022	10.1056/nejmc2202879	3
85	"We Signed Up for This!"-Student and Trainee Responses to the COVID-19 Pandemic	6/18/2020	10.1056/nejmp2005234	175
85	COVID-19 and the Mandate to Redefine Preventive Care	10/15/2020	10.1056/nejmp2018749	13
83	Remdesivir for the Treatment of COVID-19—Preliminary Report	9/3/2020	10.1056/nejmc2022236	373
78	A Trial of Lopinavir–Ritonavir in COVID-19	5/21/2020	10.1056/nejmc2008043	145
75	Scaling Up COVID-19 Vaccination in Africa—Lessons from the HIV Pandemic	7/15/2021	10.1056/nejmp2103313	11
72	Vaccinating Detained Migrants against SARS-CoV-2—Preventing Another Tragedy	1/14/2021	10.1056/nejmpv2035416	3
72	Spike D614G—A Candidate Vaccine Antigen Against COVID-19	6/17/2021	10.1056/nejmcibr2106054	5
71	Identifying and Tracking SARS-CoV-2 Variants—A Challenge and an Opportunity	7/29/2021	10.1056/nejmp2103859	11
68	Audio Interview: Efficacy of Current COVID-19 Vaccines against Variant Viruses	3/18/2021	10.1056/nejme2104584	0
66	Molnupiravir for COVID-19 in Nonhospitalized Patients	3/31/2022	10.1056/nejmc2201612	6
66	Waning mRNA-1273 Vaccine Effectiveness against SARS-CoV-2 Infection in Qatar	3/17/2022	10.1056/nejmc2119432	46
65	Effectiveness of Homologous or Heterologous COVID-19 Boosters in Veterans	4/7/2022	10.1056/nejmc2200415	20
64	Elective Surgery during the COVID-19 Pandemic	10/29/2020	10.1056/nejmclde2028735	25
63	Audio Interview: Viral Variants and COVID-19	2/18/2021	10.1056/nejme2102882	2
62	Partitioning the Curve—Interstate Travel Restrictions During the COVID-19 Pandemic	9/24/2020	10.1056/nejmp2024274	20
61	Audio Interview: COVID-19 in South Africa and a New SARS-CoV-2 Variant	1/14/2021	10.1056/nejme2100736	3
60	Case 17-2020: A 68-Year-Old Man with COVID-19 and Acute Kidney Injury	5/28/2020	10.1056/nejmcpc2002418	59
60	COVID-19 and the Investigator Pipeline	7/1/2021	10.1056/nejmp2100086	9
58	Epidemiology of COVID-19	5/7/2020	10.1056/nejmc2005157	31
57	Case 21-2020: A 66-Year-Old Homeless Man with COVID-19	7/9/2020	10.1056/nejmcpc2002421	11
52	Audio Interview: Tocilizumab and COVID-19	10/22/2020	10.1056/nejme2032051	0
52	Case 34-2022: A 57-Year-Old Woman with COVID-19 and Delusions	11/10/2022	10.1056/nejmcpc2115857	0
52	No Correlation between Anti-PF4 and Anti-SARS-CoV-2 Antibodies after ChAdOx1 nCoV-19 Vaccination	9/30/2021	10.1056/nejmc2111305	18
50	Putting the Public Back in Public Health—Surveying Symptoms of COVID-19	8/13/2020	10.1056/nejmp2016259	42
49	Genetic Risk of Severe COVID-19	10/15/2020	10.1056/nejme2025501	17
49	COVID-19 and the Safety Net—Moving from Straining to Sustaining	12/9/2021	10.1056/nejmp2114010	2
48	Audio Interview: Effectiveness of COVID-19 Vaccination in Children	3/31/2022	10.1056/nejme2204268	0
48	Choices in a Crisis—Individual Preferences among SARS-CoV-2 Vaccines	4/29/2021	10.1056/nejmp2102146	10
48	Anti-idiotype Antibodies in SARS-CoV-2 Infection and Vaccination	3/3/2022	10.1056/nejmc2119443	2
46	COVID-19-Associated Myopathy Caused by Type I Interferonopathy	12/10/2020	10.1056/nejmc2031085	52
46	COVID-19 Vaccine Trials and Incarcerated People—The Ethics of Inclusion	11/12/2020	10.1056/nejmp2025955	14
46	Case 23-2020: A 76-Year-Old Woman Who Died from COVID-19	7/23/2020	10.1056/nejmcpc2004974	14
46	Neutralizing Antibody LY-CoV555 for Outpatient COVID-19	1/14/2021	10.1056/nejmc2033787	11
45	BNT162b2 COVID-19 Vaccine in Children 5 to 11 Years of Age	2/10/2022	10.1056/nejmc2118775	4
44	Audio Interview: Combating COVID-19 Today and Tomorrow	8/11/2022	10.1056/nejme2210673	0
43	Audio Interview: What Clinicians Need to Know in Diagnosing and Treating COVID-19	3/5/2020	10.1056/nejme2004244	7
38	Nirmatrelvir for Nonhospitalized Adults with COVID-19	8/4/2022	10.1056/nejmc2206277	0
38	Audio Interview: New Research on Possible Treatments for COVID-19	3/19/2020	10.1056/nejme2005759	20
38	Audio Interview: New SARS-CoV-2 Vaccine Results, with Peter Piot	7/30/2020	10.1056/nejme2026514	1
36	BNT162b2 Vaccine Booster and COVID-19 Mortality	3/10/2022	10.1056/nejmc2120044	2
33	Audio Interview: COVID-19 and the President	10/8/2020	10.1056/nejme2031183	0
33	Audio Interview: Preparing for the Spread of COVID-19	2/27/2020	10.1056/nejme2003319	4
32	Subcutaneous REGEN-COV Antibody Combination to Prevent COVID-19	11/11/2021	10.1056/nejmc2113862	17
31	Case 22-2020: A 62-Year-Old Woman with Early Breast Cancer during the COVID-19 Pandemic	7/16/2020	10.1056/nejmcpc2002422	11
31	Audio Interview: Practical Measures to Help Prevent COVID-19	3/26/2020	10.1056/nejme2006742	4
28	A SARS-CoV-2 mRNA Vaccine-Preliminary Report	9/17/2020	10.1056/nejmc2026616	17
27	Audio Interview: Lessons from COVID-19 Hotspots	4/2/2020	10.1056/nejme2007783	3
26	Audio Interview: The Implications of Changes in the Structural Biology of SARS-CoV-2	3/11/2021	10.1056/nejme2104138	0
24	Audio Interview: The Impact of COVID-19 on Minority Communities	6/11/2020	10.1056/nejme2021935	3
24	Audio Interview: Planning for the SARS-CoV-2 Vaccine Rollout	1/7/2021	10.1056/nejme2100295	1
24	Audio Interview: Caring for Hospitalized Patients with COVID-19	12/3/2020	10.1056/nejme2034472	0
23	Baricitinib Therapy in COVID-19 Pneumonia—An Unmet Need Fulfilled	3/4/2021	10.1056/nejme2034982	45
23	Hydroxychloroquine with or without Azithromycin in COVID-19	1/14/2021	10.1056/nejmc2031780	4
23	Renin–Angiotensin–Aldosterone System Inhibitors in COVID-19	6/11/2020	10.1056/nejmc2013707	23
22	Pulmonary Vascular Pathology in COVID-19	8/27/2020	10.1056/nejmc2022068	18
22	Audio Interview: Approaches to COVID-19 Vaccines and Antivirals	4/23/2020	10.1056/nejme2012889	6
22	Plasma Therapy to Prevent Severe COVID-19 in Older Adults	6/24/2021	10.1056/nejmc2104747	17
22	Case 28-2021: A 37-Year-Old Woman with COVID-19 and Suicidal Ideation	9/16/2021	10.1056/nejmcpc2107350	1
22	Personal Protective Equipment and COVID-19	7/23/2020	10.1056/nejmc2021986	1
21	Audio Interview: The Real-World Effectiveness of COVID-19 Vaccination	2/25/2021	10.1056/nejme2103272	0
21	More on Clinical Characteristics of Pregnant Women with COVID-19 in Wuhan, China	8/13/2020	10.1056/nejmc2016881	6
20	Audio Interview: COVID-19 Vaccine Development	7/16/2020	10.1056/nejme2025293	0
20	Audio Interview: Are COVID-19 Vaccine Boosters Necessary?	10/7/2021	10.1056/nejme2115835	0
20	Audio Interview: Understanding Antibody Testing in COVID-19	9/3/2020	10.1056/nejme2028992	1
20	Audio Interview: Caring for Patients with COVID-19	4/16/2020	10.1056/nejme2011242	1
19	More on SARS-CoV-2 Infection after Vaccination in Health Care Workers	7/8/2021	10.1056/nejmc2106004	4
19	Intrinsic Severity of the SARS-CoV-2 Omicron Variant	5/12/2022	10.1056/nejmc2203679	0
19	Audio Interview: Dexamethasone and COVID-19	7/23/2020	10.1056/nejme2025927	2
18	Tocilizumab in COVID-19	1/7/2021	10.1056/nejmc2032911	14
18	Audio Interview: Understanding the Omicron Variant of SARS-CoV-2	2/24/2022	10.1056/nejme2202699	4
18	Audio Interview: A COVID-19-Related Syndrome in Children	7/2/2020	10.1056/nejme2024117	3
17	More on Neurologic Features in Severe SARS-CoV-2 Infection	6/25/2020	10.1056/nejmc2015132	19
17	Audio Interview: Delivering COVID-19 Vaccines to Minority Communities	4/1/2021	10.1056/nejme2105496	1
16	Interleukin-6 Receptor Antagonists in Critically Ill Patients with COVID-19	9/16/2021	10.1056/nejmc2108482	28
16	Audio Interview: A New Antiviral against COVID-19	2/17/2022	10.1056/nejme2202349	0
16	Audio Interview: SARS-CoV-2 Vaccination and Vulnerable Populations	12/10/2020	10.1056/nejme2034906	1
16	Audio Interview: New Evidence on SARS-CoV-2 Vaccine Boosters	9/16/2021	10.1056/nejme2115200	0
16	More on COVID-19 in Immune-Mediated Inflammatory Diseases	8/20/2020	10.1056/nejmc2018011	6
15	Early Treatment with Sotrovimab for COVID-19	4/14/2022	10.1056/nejmc2201606	1
15	Audio Interview: Waning Immunity against COVID-19	5/26/2022	10.1056/nejme2206981	0
15	BNT162b2 COVID-19 Vaccine in Adolescents	9/30/2021	10.1056/nejmc2113394	5
15	Audio Interview: Operation Warp Speed and COVID-19 Therapeutics	9/17/2020	10.1056/nejme2029886	0
15	Audio Interview: Loosening COVID-19 Restrictions	4/30/2020	10.1056/nejme2014793	6
15	Protection against SARS-CoV-2 after Vaccination and Previous Infection	6/30/2022	10.1056/nejmc2205618	4
15	Allocating Medical Resources in the Time of COVID-19	5/28/2020	10.1056/nejmc2009666	19
14	Audio Interview: Can Vaccines Prevent Transmission of COVID-19?	9/22/2022	10.1056/nejme2212576	0
14	Audio Interview: The Effects of COVID-19 on Children	4/21/2022	10.1056/nejme2205254	0
14	Audio Interview: The Legal Basis for COVID-19 Restrictions and Mandates	11/18/2021	10.1056/nejme2118197	0
13	Audio Interview: How Much Protection Does Prior SARS-CoV-2 Infection Provide?	12/16/2021	10.1056/nejme2119539	0
13	Fourth Dose of BNT162b2 mRNA COVID-19 Vaccine	7/14/2022	10.1056/nejmc2206926	1
12	Audio Interview: COVID-19 Vaccine Fundamentals	12/17/2020	10.1056/nejme2035370	2
12	Audio Interview: Communicating COVID-19 Science	4/28/2022	10.1056/nejme2205606	0
12	Maintaining Safety with SARS-CoV-2 Vaccines	3/11/2021	10.1056/nejmc2100766	9
12	Audio Interview: Acute Lung Injury in COVID-19	7/9/2020	10.1056/nejme2024719	0
12	Audio Interview: A New Look at COVID-19 Vaccine Boosters	10/21/2021	10.1056/nejme2116820	0
11	Audio Interview: Building a Successful Public Health Response to COVID-19	8/13/2020	10.1056/nejme2027574	0
11	Outpatient Remdesivir to Prevent Progression to Severe COVID-19	3/17/2022	10.1056/nejmc2200591	6
11	Lupus Anticoagulant in Patients with COVID-19	11/5/2020	10.1056/nejmc2027508	5
10	Audio Interview: COVID-19 as an Endemic Disease	2/10/2022	10.1056/nejme2201982	0
10	Audio Interview: New Studies of COVID-19 Transmission	11/26/2020	10.1056/nejme2034094	0
10	Audio Interview: A New Monoclonal Antibody for COVID-19	10/29/2020	10.1056/nejme2032410	2
10	Tocilizumab for COVID-19—The Ongoing Search for Effective Therapies	12/10/2020	10.1056/nejme2032071	29
10	Audio Interview: A Look at the U.S. Government's COVID-19 Strategy	7/21/2022	10.1056/nejme2209791	0
10	Effectiveness of an Inactivated SARS-CoV-2 Vaccine	9/30/2021	10.1056/nejmc2112423	4
10	Convalescent Plasma for COVID-19—Making Sense of the Inconsistencies	5/5/2022	10.1056/nejme2204332	4
9	Audio Interview: Studying Potential COVID-19 Therapies	5/7/2020	10.1056/nejme2015955	2
9	Audio Interview: Waning Immunity against SARS-CoV-2	12/9/2021	10.1056/nejme2119211	1
9	Screening for COVID-19 in Skilled Nursing Facilities	7/9/2020	10.1056/nejmc2017362	2
9	Audio Interview: COVID-19 and Contact Tracing	8/20/2020	10.1056/nejme2028055	1
9	Viable SARS-CoV-2 Shedding	4/29/2021	10.1056/nejmc2102494	4
9	Audio Interview: Finding Reliable Information about COVID-19	5/14/2020	10.1056/nejme2017594	0
8	Audio Interview: COVID-19 in Children	6/17/2021	10.1056/nejme2110211	0
8	An Outbreak of COVID-19 on an Aircraft Carrier	3/11/2021	10.1056/nejmc2034424	13
8	Audio Interview: The Impact of COVID-19 on Patients with Other Diseases, with Arnold Epstein	8/6/2020	10.1056/nejme2027046	2
8	Audio Interview: Vaccinology and COVID-19	10/15/2020	10.1056/nejme2031646	0
8	Audio Interview: Developing New COVID-19 Vaccines	10/13/2022	10.1056/nejme2213420	0
7	First Case of COVID-19 in the United States	5/7/2020	10.1056/nejmc2004794	13
7	Audio Interview: What Earlier Epidemics Teach Us about COVID-19	3/25/2021	10.1056/nejme2105030	1
7	Rapid Decay of Anti-SARS-CoV-2 Antibodies in Persons with Mild COVID-19	9/10/2020	10.1056/nejmx200017	14
7	Audio Interview: COVID-19 Vaccines and the FDA	7/14/2022	10.1056/nejme2209406	0
7	Audio Interview: COVID-19 in Europe and New Information on Vaccines	11/19/2020	10.1056/nejme2033666	0
7	Audio Interview: Guidelines for COVID-19 Vaccine Deployment	9/10/2020	10.1056/nejme2029435	1
7	RAAS Inhibitors and Risk of COVID-19	11/12/2020	10.1056/nejmc2030446	14
6	Audio Interview: New Data on Remdesivir in COVID-19	5/28/2020	10.1056/nejme2019975	1
6	Audio Interview: Protecting the Immunosuppressed against COVID-19	5/27/2021	10.1056/nejme2108949	0
6	Audio Interview: Diagnosis and Early Treatment of COVID-19	6/4/2020	10.1056/nejme2021023	0
6	Audio Interview: What's Gone Right in Our Battle against COVID-19	11/25/2021	10.1056/nejme2118514	1
5	Audio Interview: A New Round of Rising COVID-19 Numbers	9/2/2021	10.1056/nejme2114471	0
5	Audio Interview: Designing the Next COVID-19 Vaccine	7/7/2022	10.1056/nejme2209134	0
5	Monoclonal Antibody for Patients with COVID-19	3/25/2021	10.1056/nejmc2100221	1
5	Audio Interview: A Look at SARS-CoV-2 Transmission	6/18/2020	10.1056/nejme2022576	0
4	Audio Interview: Updated COVID-19 Vaccines and a Look at Monkeypox	8/4/2022	10.1056/nejme2210394	0
4	Tracking the Emergence of SARS-CoV-2 Alpha Variant in the United Kingdom	12/30/2021	10.1056/nejmc2103227	19
4	Audio Interview: Protecting the Immunocompromised from COVID-19	6/9/2022	10.1056/nejme2207596	0
4	Audio Interview: COVID-19 and the Media	9/30/2021	10.1056/nejme2115825	0
4	Audio Interview: COVID-19 Testing and the Individual Physician	10/1/2020	10.1056/nejme2030753	0
4	Audio Interview: COVID-19: Where Are We Heading?	10/20/2022	10.1056/nejme2213773	0
4	Audio Interview: Choosing an Antiviral to Treat COVID-19	8/25/2022	10.1056/nejme2211254	0
4	Audio Interview: COVID-19 in Brazil and New Evidence for Vaccinating Younger Children	11/11/2021	10.1056/nejme2117854	1
4	Audio Interview: Advice for Clinicians on COVID-19 Vaccines and Social Restrictions	4/29/2021	10.1056/nejme2107327	2
4	Audio Interview: The Impact of COVID-19 on Trainees and Junior Faculty	7/22/2021	10.1056/nejme2112375	1
4	Audio Interview: Assessing Bivalent Vaccines against COVID-19	9/29/2022	10.1056/nejme2212736	0
4	Audio Interview: How Effective is COVID-19 Vaccination in Children?	6/30/2022	10.1056/nejme2208670	0
3	Audio Interview: Caring for Hospitalized Patients with COVID-19	11/3/2022	10.1056/nejme2214245	0
3	Audio Interview: Capitalizing on Immune Responses to COVID-19	5/21/2020	10.1056/nejme2019020	0
3	Convalescent Plasma for COVID-19	9/8/2022	10.1056/nejmc2208338	0
3	Audio Interview: COVID-19 and the WHO	9/1/2022	10.1056/nejme2211564	0
3	Audio Interview: Do We Have the Tools to End the COVID-19 Pandemic?	11/4/2021	10.1056/nejme2117543	0
3	Audio Interview: How Well Are COVID-19 Vaccines Working?	7/8/2021	10.1056/nejme2111369	1
3	Audio Interview: COVID-19 Vaccination and the Omicron Variant	12/30/2021	10.1056/nejme2120098	0
3	Audio Interview: Vaccine Efficacy and Boosters in COVID-19	9/23/2021	10.1056/nejme2115556	0
3	Audio Interview: COVID-19 and the States—A Conversation with Ralph Northam	2/4/2021	10.1056/nejme2102043	0
3	The COVID-19 Vaccine-Development Multiverse	2/18/2021	10.1056/nejmc2034838	2
3	Audio Interview: Addressing the Omicron Variant of SARS-CoV-2	1/27/2022	10.1056/nejme2201214	0
3	Audio Interview: Rolling Out New COVID-19 Vaccines	9/8/2022	10.1056/nejme2211875	0
3	Audio Interview: Do We Need New COVID-19 Vaccines?	5/5/2022	10.1056/nejme2205974	1
3	Audio Interview: COVID-19—Why We Publish What We Do	3/24/2022	10.1056/nejme2203970	0
3	Audio Interview: A New Monoclonal Antibody for COVID-19 and Potential Vaccination for Children	10/28/2021	10.1056/nejme2117187	0
3	Audio Interview: Reporting on COVID-19	10/14/2021	10.1056/nejme2115837	0
3	SARS-CoV-2 Vaccine-Induced Immune Thrombotic Thrombocytopenia	4/20/2021	10.1056/nejmx210006	4
3	Audio Interview: A New SARS-CoV-2 Vaccine and a New Look at Treatment	3/4/2021	10.1056/nejme2103680	0
3	Audio Interview: A Look at COVID-19 Prevention and Care in 2020	12/31/2020	10.1056/nejme2036225	1
2	Protection Due to Previous SARS-CoV-2 Infection	6/23/2022	10.1056/nejmc2205555	0
2	Audio Interview: Can We Make More Effective COVID-19 Vaccines?	3/17/2022	10.1056/nejme2203667	0
2	COVID-19 Infections in Vaccinated Health Care Workers	1/13/2022	10.1056/nejmc2117817	0
2	Saliva for Detection of SARS-CoV-2	3/4/2021	10.1056/nejmc2032165	2
2	The Future of SARS-CoV-2 Vaccination	3/3/2022	10.1056/nejmc2119437	3
2	Convalescent Plasma for Outpatients with COVID-19	12/2/2021	10.1056/nejmc2114591	1
2	Audio Interview: Developing Mucosal Immunity to COVID-19	9/15/2022	10.1056/nejme2212241	0
2	Audio Interview: Crushing the COVID-19 Curve	1/20/2022	10.1056/nejme2200865	0
2	SARS-CoV-2 Infection among Nursing Home Residents	11/11/2021	10.1056/nejmc2114113	1
2	Audio Interview: Another New COVID-19 Vaccine	7/1/2021	10.1056/nejme2110939	0
2	Audio Interview: An International Look at COVID-19	1/21/2021	10.1056/nejme2101204	0
2	Audio Interview: Operation Warp Speed and COVID-19	8/27/2020	10.1056/nejme2028547	0
2	Audio Interview: Vaccinating Young Children against COVID-19	6/23/2022	10.1056/nejme2208292	0
2	Audio Interview: Dissecting the Host Response to SARS-CoV-2	6/2/2022	10.1056/nejme2207246	0
2	Early Spread of SARS-CoV-2 in the Icelandic Population	11/26/2020	10.1056/nejmc2027653	18
2	Audio Interview: Eight Months of Action and Inaction against COVID-19	9/24/2020	10.1056/nejme2030336	0
1	Audio Interview: What to Make of COVID-19 Hot Spots	5/19/2022	10.1056/nejme2206659	0
1	Audio Interview: Using Our COVID-19 Experience to Develop Vaccines More Quickly	3/3/2022	10.1056/nejme2203035	0
1	Audio Interview: COVID-19 Takeaways at Podcast 100	1/13/2022	10.1056/nejme2200490	0
1	Audio Interview: Aspects of COVID-19 Immunity	8/19/2021	10.1056/nejme2113747	2
1	Tocilizumab in Patients Hospitalized with COVID-19 Pneumonia	4/15/2021	10.1056/nejmc2100217	17
1	Audio Interview: The Legal Basis for COVID-19 Restrictions and Mandates.	11/18/2021	10.1056/nejme2118917	0
1	Rapid Diagnostic Testing for SARS-CoV-2	4/28/2022	10.1056/nejmc2202308	1
1	SARS-CoV-2 Human Challenge Studies	10/28/2021	10.1056/nejmc2113574	0
1	Surviving COVID-19 with Heparin?	9/9/2021	10.1056/nejmx210013	1

## References

[1] (2022). Altmetric. Guide for describing Altmetric data in publications. Published.

[2] (2022). Altmetric. The most discussed and shared research and commentary of 2020. https://www.altmetric.com/top100/2020/press-release.pdf.

[3] Walker M, Katerina M (2021). News Consumption Across Social Media in 2021. Published.

[4] (2022). Altmetric. How is the Altmetric attention score calculated?. https://help.altmetric.com/support/solutions/articles/6000233311-how-is-the-altmetric-attention-score-calculated-#:~:text=TheAltmetricAttentionScorefor,attentionthatithasreceived.&text=Thescoreisderivedfrom,upforaresearchoutput.

[5] Patthi B, Prasad M, Gupta R (2017). Altmetrics-a collated adjunct beyond citations for scholarly impact: a systematic review. J Clin Diagn Res.

[6] Amath A, Ambacher K, Leddy JJ, Wood TJ, Ramnanan CJ (2017). Comparing alternative and traditional dissemination metrics in medical education. Med Educ.

[7] Rosenkrantz AB, Ayoola A, Singh K, Duszak R Jr (2017). Alternative metrics ("Altmetrics") for assessing article impact in popular general radiology journals. Acad Radiol.

[8] Asaad M, Howell SM, Rajesh A, Meaike J, Tran NV (2020). Altmetrics in plastic surgery journals: does it correlate with citation count?. Aesthet Surg J.

[9] Barakat AF, Nimri N, Shokr M, Mahtta D, Mansoor H, Masri A, Elgendy IY (2019). Correlation of altmetric attention score and citations for high-impact general medicine journals: a cross-sectional study. J Gen Intern Med.

[10] Chen WM, Bukhari M, Cockshull F, Galloway J (2020). The relationship between citations, downloads and alternative metrics in rheumatology publications: a bibliometric study. Rheumatology (Oxford).

[11] Costas R, Zahedi Z, Wouters P (2015). Do “altmetrics” correlate with citations? Extensive comparison of altmetric indicators with citations from a multidisciplinary perspective. J Assoc Inf Sci Technol.

[12] Didegah F, Bowman TD, Holmberg K (2018). On the differences between citations and altmetrics: an investigation of factors driving altmetrics versus citations for Finnish articles. J Assoc Inf Sci Technol.

[13] Ayoub F, Ouni A, Case R, Ladna M, Shah H, Rubin DT (2021). Dissemination of gastroenterology and hepatology research on social media platforms is associated with increased citation count. Am J Gastroenterol.

[14] Lamb CT, Gilbert SL, Ford AT (2018). Tweet success? Scientific communication correlates with increased citations in Ecology and Conservation. PeerJ.

[15] Giustini AJ, Axelrod DM, Lucas BP, Schroeder AR (2020). Association between citations, altmetrics, and article views in pediatric research. JAMA Netw Open.

[16] Huang A, Lee AG, Weng CY (2022). Altmetric attention scores in ophthalmology journals. JAMA Ophthalmol.

[17] Shrivastava R, Mahajan P (2016). Relationship between citation counts and Mendeley readership metrics. New Libr World.

[18] Thelwall M (2018). Early Mendeley readers correlate with later citation counts. Scientometrics.

